# Diabetic Striatopathy: An Extremely Rare Form of Diabetes Mellitus End-Organ Lesions

**DOI:** 10.7759/cureus.74554

**Published:** 2024-11-27

**Authors:** Luis H Luz, Sandra Cunha, Cláudia Diogo, Nádia Santos, Ana U Ferrão

**Affiliations:** 1 Internal Medicine, Hospital Santo André, Unidade Local de Saúde da Região de Leiria, Leiria, PRT; 2 Internal Medicine, Unidade Local de Saúde da Região de Leiria, Leiria, PRT

**Keywords:** chorea, diabetes mellitus, hyperglycemia, neuropathy, striatopathy

## Abstract

Diabetes mellitus is one of the most frequent endocrinopathies in the medical routine, appearing across different specialties. Although neurological involvement in the form of peripheral neuropathy is the most recurrent form acknowledged by physicians, the spectrum of neurological involvement can be more diverse. Here, we present a case of diabetic striatopathy, a rare neurological manifestation of diabetes mellitus with poor metabolic control, in a patient whose epidemiological group was not classically. As clinical and imaging findings are typical, although rare, a high degree of suspicion is necessary for diagnosis. This case report discusses the clinical findings, radiological workup, and therapeutic measures needed for the management of this disease.

## Introduction

Diabetic striatopathy, also known as non-ketotic hyperglycemic hemichorea or chorea hyperglycemia basal ganglia syndrome, is a rare neurological syndrome and a manifestation of the neurological consequences of poorly controlled diabetes mellitus. Diabetic striatopathy is part of the spectrum of hyperosmolar manifestations of diabetes mellitus together with the hyperglycemic hyperosmolar state [1].

## Case presentation

A 78-year-old Caucasian female presented to the emergency department with a five-day onset of facial palsy and left arm weakness. The patient had known diagnoses of hypertension, dyslipidemia, depression, and type 2 diabetes mellitus with erratic therapeutic compliance. At admission, the patient was dehydrated, and vital signs revealed normotension but a heart rate of 107 beats/minute. Point-of-care blood glucose level was 379 mg/dL. A neurological examination revealed left central facial palsy, with chorea affecting only the left superior limb. A blood workup confirmed high glucose levels (Table 1).

**Table 1:** Analytical findings noted during the initial evaluation.

Parameter	Value	Normal range
Hemoglobin	14.7 g/dL	11.5–16 g/dL
Leukocytes	13.9 g/dL	4–10 g/dL
C-reactive protein	45 mg/L	<5.0 mg/L
Urea	88 mg/dL	17–43 mg/L
Creatinine	1.47 mg/dL	0.51–0.95 mg/dL
Sodium	132 mmol/L	136–146 mmol/L
Potassium	3.5 mmol/L	4.5–5.1 mmol/L
Serum glucose	339 mg/dL	74–106 mg/dL

As arterial blood gas analysis did not reveal acidemia and ketone bodies were not detected, the diagnosis of diabetic ketoacidosis was ruled out. The presence of hyperglycemia and hyperosmolarity are similar to the criteria for a hyperosmolar hyperglycemic state, but capillary blood glucose showed a value below 600 mg/dL.

**Figure 1:**
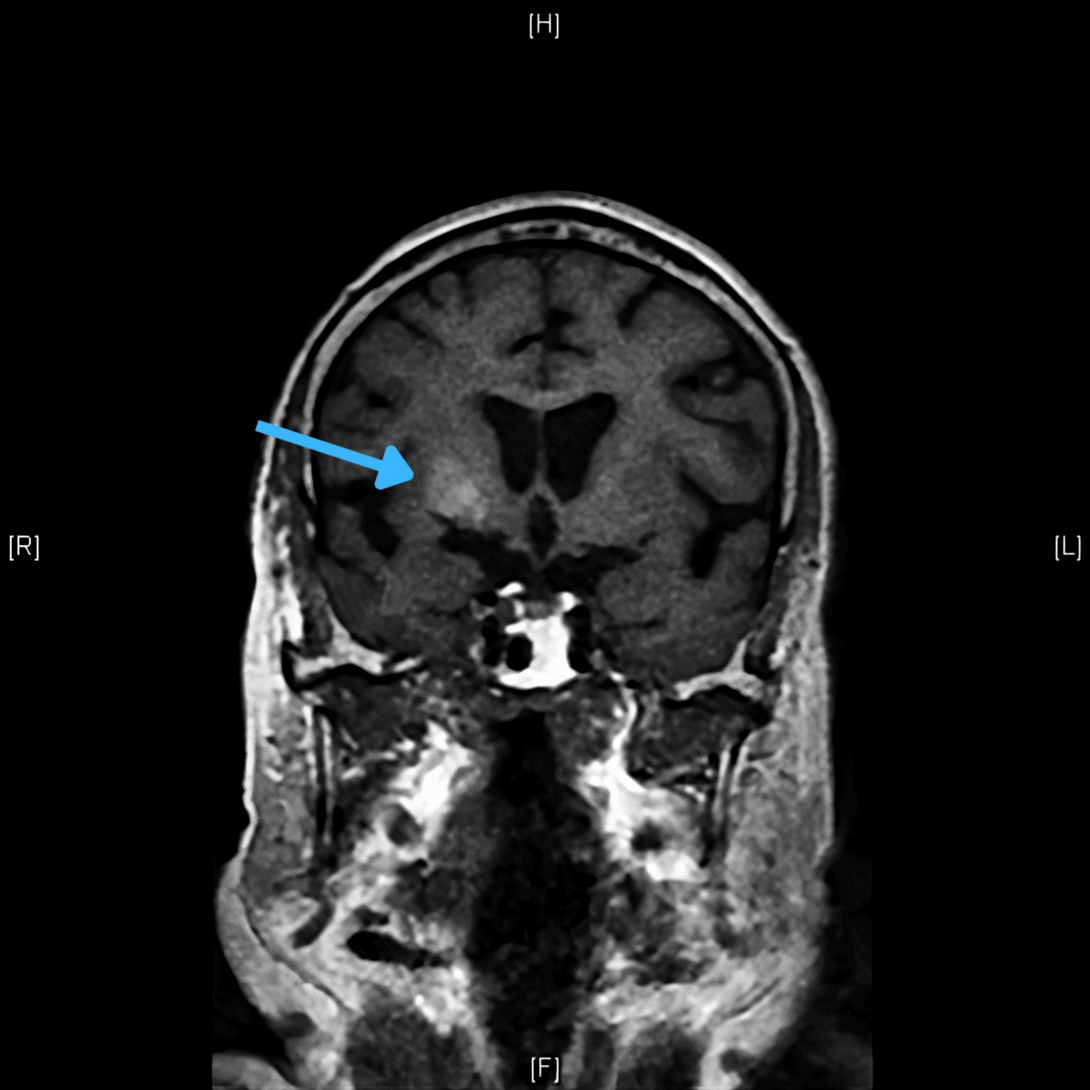
MRI with frontal view of T1 weighting highlighting spontaneous hypersignal in the affected basal ganglia (contralateral to the choreiform limb).

**Figure 2:**
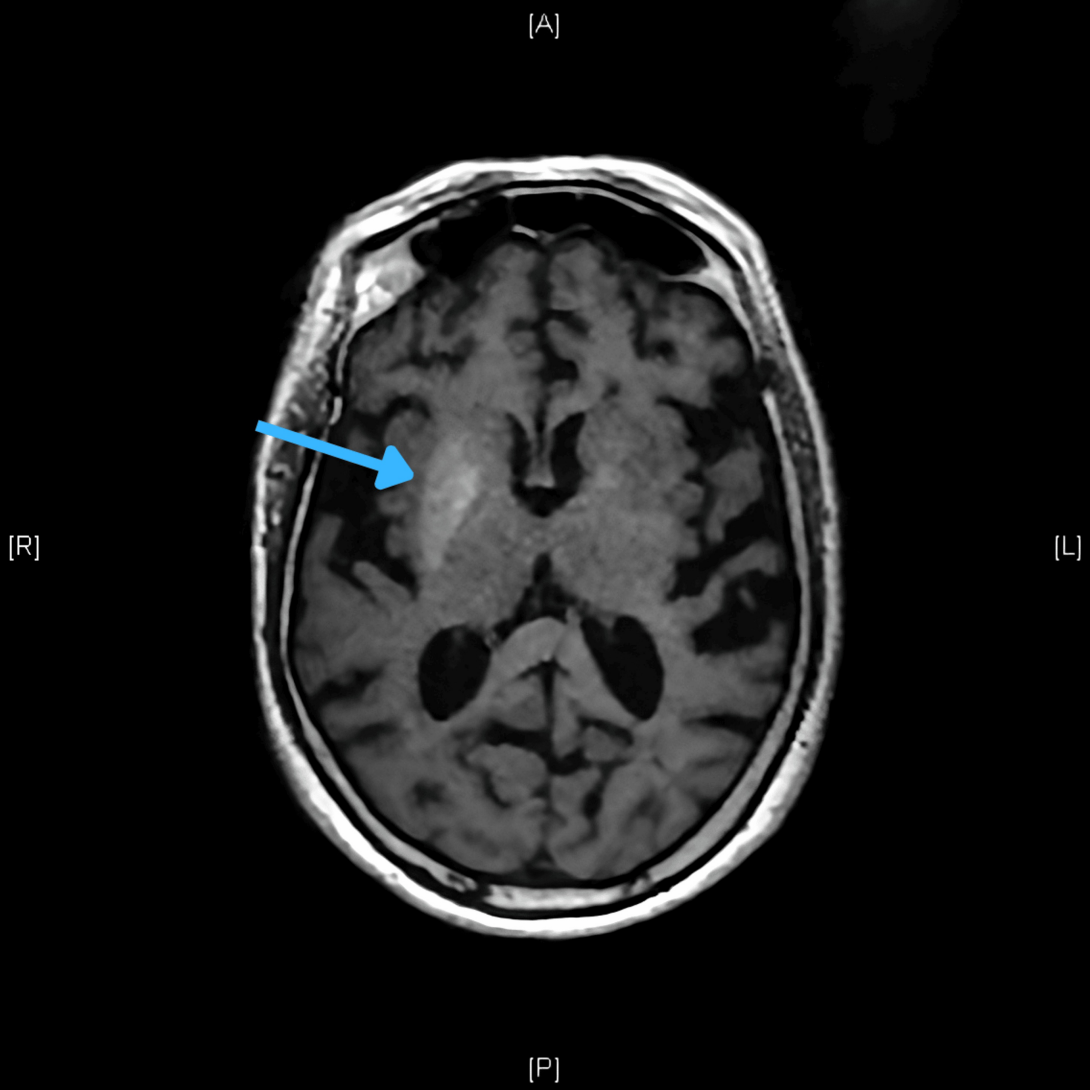
MRI with an axial view of T1 weighting highlighting spontaneous hypersignal in the affected basal ganglia (contralateral to the choreiform limb).

The remaining workup was unremarkable, except for a CT scan of the brain, which showed spontaneous hyperdensity on the right striatum. MRI performed subsequently confirmed a high T1 signal in the same area, establishing the diagnosis of diabetic striatopathy (Figures 1, 2).

The patient was started on intravenous insulin infusion until capillary blood glucose levels stabilized. Serial monitoring of glucose levels and serum electrolytes was conducted throughout this period. Once glycemic control was achieved, a weight-adjusted daily dose of long-acting insulin was administered, and systematic blood glucose corrections with rapid-acting insulin were scheduled at four-hour intervals to ensure continued stabilization. Intravenous hydration was maintained throughout the surveillance. Control of choreiform movements was attempted using injectable haloperidol but without success. Surveillance was done in a dedicated ward and progressive improvement and gradual resolution of symptoms were observed only after glycemic control was achieved.

## Discussion

Stephen F. Bedwell first described this entity in 1960 [2]. Diabetic striatopathy is a rare clinical syndrome with an estimated prevalence of around 1:100,000 individuals [3], and it is believed to be underestimated. It is more frequently observed in females with a greater predominance in the Asian population (71.6% of known cases) with an average age of onset of 67.6 years [4]. The presented case is interesting as the patient was a nearly 80-year-old Caucasian individual, contrasting with the usually afflicted group.

Chorea is defined as a hyperkinetic dyskinesia with involuntary, unpredictable arrhythmic movements, with low amplitude and distal predominance. On the other hand, hemiballismus is described as an involuntary movement of large amplitude, arrhythmic, and predominantly proximal. Chorea and hemiballismus are frequent manifestations of diabetic striatopathy, being reported in about 97.7% of cases according to Chua et al. [5]. Both types of dyskinesia are more pronounced during periods of wakefulness and may cease during sleep. Anxiety and emotional stress often exacerbate the condition [4]. The intensity and unpredictability of movements may represent an increased risk of trauma inflicted on oneself and others.

The underlying pathophysiological mechanism through which hyperglycemia induces damage to the striatum is not completely understood. However, some pathophysiological models try to justify it. The hyperglycemic state observed in patients with uncontrolled diabetic mellitus seems to induce dysfunction of brain autoregulation mechanisms. The state of intravascular hyperosmolarity and hyperviscosity generated by hyperglycemia leads to blood-brain barrier dysfunction and ischemic injury at the level of the putamen.

Moreover, the induction of anaerobic metabolism depletes gamma-aminobutyric acid, whose importance is linked to its role as a neurotransmitter with inhibitory activity. The consequent disinhibition of thalamocortical pathways results in cortical hyperexcitability, manifesting as dyskinesia. The combination of vascular and metabolic injury culminates in transient dysfunction of the thalamic apparatus, resulting in the clinical picture explored above.

Cases of diabetic striatopathy have been described in normoglycemic individuals presenting in the emergency department. However, HbA1c characterization invariably reveals poor baseline metabolic control.

The etiologies of chorea and hemiballismus are vast. Non-ketonic hyperglycemia constitutes the most frequent etiology (among the metabolic causes), accounting, however, for only 1% of the causes of chorea and hemiballismus [6].

The diagnostic criteria should include a comprehensive anamnesis and a rigorous neurological examination that allows the exclusion of other neurological entities that co-occur with dyskinetic conditions. It is important to consider all major groups of pathologies, namely, vascular disease, metabolic disorders, diverse types of central nervous system infections, and autoimmune abnormalities, as some of the most over-presented causes. However, the combination of a compatible anamnesis, physical examination, and personal history, associated with typical imagological findings on CT or MRI, allow for a faster and more targeted diagnostic process. Diabetic striatopathy presents with typical and highly suggestive alterations on CT and MRI.

MRI proved to be the test with the highest sensitivity for detecting changes induced by hyperglycemia in the striatum region, with the putamen usually being the most frequently involved basal ganglia [4]. A suggestive image of a metabolic disorder of the striatum apparatus consists of a hyperdense lesion on CT, or hyperintense on T1-weighted MRI (without mass effect or edema) located in the basal ganglia contralateral to the affected limbs. On MRI, T2-weighted/fluid-attenuated inversion recovery images may show variable features but usually demonstrate hypointensity of the basal ganglia. Diffusion-weighted images do not show diffusion restriction [1,4]. The documentation of a T1 hypersignal image located at the basal ganglia should motivate a differential diagnosis with entities such as Wilson’s disease, or a possible striatocapsular infarction.

On CT, the hyperdensity of this anatomic structure should highlight the possibility of a hemorrhagic stroke, calcification, Tay-Sachs disease, and tuberous sclerosis [1]. The unavailability of complementary study methods with a functional component (functional MRI) makes their use in emergencies unfeasible. In some cases, it is possible to detect signal alteration that extends from the globus pallidus through the nigrostriatal tract.

MRI with spectroscopy may reveal low N-acetyl aspartate-creatinine ratios and high choline-creatinine ratios. Positron emission tomography scans may demonstrate reduced metabolic rate and glucose consumption in the affected basal ganglia. Histologically, the described imaging changes translate into a loss of neuronal density, gliosis, and astrocyte proliferation [7,8]. High diagnostic suspicion and the recognition of indicative imaging patterns are crucial for early recognition and prompt institution of corrective measures.

The condition’s responsiveness to glycemic correction is high, but imaging changes may be more persistent, remaining detectable for months or even years after the acute condition [9]. Glycemic control is the cornerstone of the therapeutic approach. The treatment plan should include non-pharmacological and pharmacological measures for the parallel management of the underlying metabolic dysfunction and consequences arising from dyskinesia.

The implementation of antidiabetic therapy is essential for controlling the underlying pathophysiology. This measure is essential, both in patients who present with high blood glucose and in patients with normal glycemia at the time of observation but with high HbA1c, reflecting poor prior glycemic control. Diet review, physical exercise, sleep regulation, and other aspects that influence the cardiovascular risk profile should be revisited. It is equally beneficial to dedicate some time to psychoeducation to reduce the stigma and motivate a change in therapeutic compliance. At the same time, the management of the motor condition and potential harmful consequences for oneself and third parties requires, in itself, pharmacological control measures.

Antipsychotics have been applied in the acute management of this syndrome. However, they should not be used without due consideration of the adverse effects, such as metabolic syndrome, dysphoria, and a punctual increase in the risk of mortality, especially in elderly groups. Therapeutic alternatives are benzodiazepines (clonazepam) and dopamine receptor antagonists such as olanzapine, tetrabenazine, and tiapride [5].

The reassurance and explanation of the baseline condition may attenuate the anxiogenic component, in itself a reason for accentuating the dyskinetic condition. The prognosis of the condition is generally favorable. Responsiveness to therapeutic measures is usually high, and the benefits of glycemic control are observable in the short term.

## Conclusions

Diabetic striatopathy is a rare end-organ manifestation in the context of diabetes mellitus with poor glycemic control. The condition and the underlying risk of self-harm dictate the initial approach in an emergency context. Acute hyperglycemia is not mandatory for the development of dyskinetic symptoms in diabetic striatopathy. The alternative diagnoses are vast and must be considered, but a high level of suspicion together with the typical imaging characterization allows a timely diagnosis and the immediate institution of treatment measures. If diabetic striatopathy is suspected, the appearance of hyperintense images on MRI should require a differential diagnosis of intracranial hemorrhage. Adequate glycemic control is the cornerstone of the therapeutic approach.
